# *PLP1*-Associated Demyelinating Neuropathy in 2 Female Patients

**DOI:** 10.1212/NXG.0000000000200441

**Published:** 2026-11-03

**Authors:** Anderson Brito da Silva, Marian Nicolas Vela, Ming Lai, Lelia Vico Pardo, Amal P.R. Samaraweera, Rita Horvath, Patrick F. Chinnery, Mark Roger Baker, Michael John Keogh

**Affiliations:** 1 Translational and Clinical Research Institute, Newcastle University, Newcastle Upon Tyne, United Kingdom;; 2 Department of Clinical Neurophysiology, Royal Victoria Infirmary, Newcastle Upon Tyne, United Kingdom;; 3 Department of Clinical Neurophysiology, Hospital Universitario Virgen de las Nieves, Granada, Spain;; 4 Department of Neurology, Royal Victoria Infirmary, Newcastle Upon Tyne, United Kingdom;; 5 Department of Clinical Neurosciences, University Neurology Unit, Cambridge Biomedical Campus, United Kingdom; and; 6 Biosciences Institute, The Medical School, Newcastle University, Newcastle Upon Tyne, United Kingdom.

## Abstract

**Objective:**

The aim of this study was to report 2 unrelated women with demyelinating motor-predominant neuropathy in whom pathogenic variants in the proteolipid protein 1 gene (*PLP1*) were identified.

**Methods:**

Two unrelated female individuals underwent nerve conduction studies, EMG, evoked potential tests, neuroimaging, and targeted genetic testing.

**Results:**

The first patient, a 34-year-old woman, presented with foot drop and had electroneurographic findings consistent with a demyelinating motor-predominant neuropathy, with genetic testing revealing a pathogenic *PLP1* variant. The second patient, also middle-aged, had a different pathogenic *PLP1* variant and a clinical diagnosis of progressive spastic paraplegia, with detailed electrophysiologic assessment demonstrating a coexistent demyelinating motor-predominant neuropathy.

**Discussion:**

These cases demonstrate that *PLP1*-related disease in women can present in the fourth and fifth decades as a sporadic demyelinating motor-predominant neuropathy, which may be mistaken for an acquired immune-mediated neuropathy. In middle-aged women presenting with an apparently sporadic demyelinating motor neuropathy, genetic testing should, therefore, include *PLP1*. Recognizing *PLP1* disease has important implications, such as access to emerging genetic therapies and the opportunity to offer preimplantation genetic testing to reduce the risk of transmission to offspring.

## Introduction

Sporadic demyelinating motor neuropathy presenting in the fourth and fifth decades with distal lower limb weakness would typically be considered an acquired immune-mediated disorder, such as motor-predominant chronic immune-mediated demyelinating polyradiculoneuropathy (CIDP) or multifocal motor neuropathy. Genetic causes in such a scenario are recognized but rare and frequently overlooked.^1^

We describe a 34-year-old woman who presented with foot drop. Electroneurographic findings were consistent with a demyelinating motor-predominant neuropathy, and she was found to carry a variant in the proteolipid protein 1 gene (*PLP1*). We also describe a second unrelated female patient with a separate *PLP1* variant and a diagnosis of progressive spastic paraplegia, in whom a coexistent demyelinating motor-predominant neuropathy was also identified.

We propose that pathologic *PLP1* variants should be considered in female individuals presenting with demyelinating motor-predominant neuropathy. *PLP1* testing is not included in most genetic diagnostic panels for hereditary neuropathies and, therefore, may be an underappreciated cause of this presentation.

## Methods

In both cases, nerve conduction studies and EMG were performed using a Micromed EMG system. The focused study protocol, adapted by the responsible clinical neurophysiologist, included bilateral upper and lower limb assessments. eMethods provides further details.

### Standard Protocol Approvals, Registrations, and Patient Consents

All investigations and tests reported were performed as part of routine clinical care. All data were acquired with informed consent and retained according to the General Data Protection Regulation (GDPR) and Caldicott Principles. Historic data presented herein have been anonymized. Reporting of anonymized historic case series does not require formal review and approval by National Health Service (NHS) Research Ethics Committees, as confirmed by the NHS Health Research Authority. Written informed consent for publication of the clinical details was obtained from both patients.

### Data Availability

The data supporting the reported findings comprise routine clinical neurophysiology and imaging records stored within a secure electronic medical record system at our institution. These data are not publicly accessible and cannot be shared because of patient confidentiality and local information governance regulations.

## Results

### Patient 1

A 34-year-old woman presented with a 6–8-month history of catching her right foot and occasional but increasingly frequent falls. There were no sensory symptoms, including pain.

The patient had a medical history of epilepsy with focal dyscognitive seizures (seizure-free for ∼12 years on lamotrigine monotherapy). She reported normal development and attended mainstream school. There was no psychiatric history.

She had 4 brothers with a diagnosis of Pelizaeus-Merzbacher disease (PMD) due to a hemizygous frameshift variant (c.49_52delGCTT, p Ala17Profs*29) in exon 2 of the *PLP1* gene (NM_000533.5; Figure). Their mother was the carrier of this variant and developed a mild spastic paraplegia (with normal nerve conduction studies) in her 60s as previously reported.^1^

**Figure:**
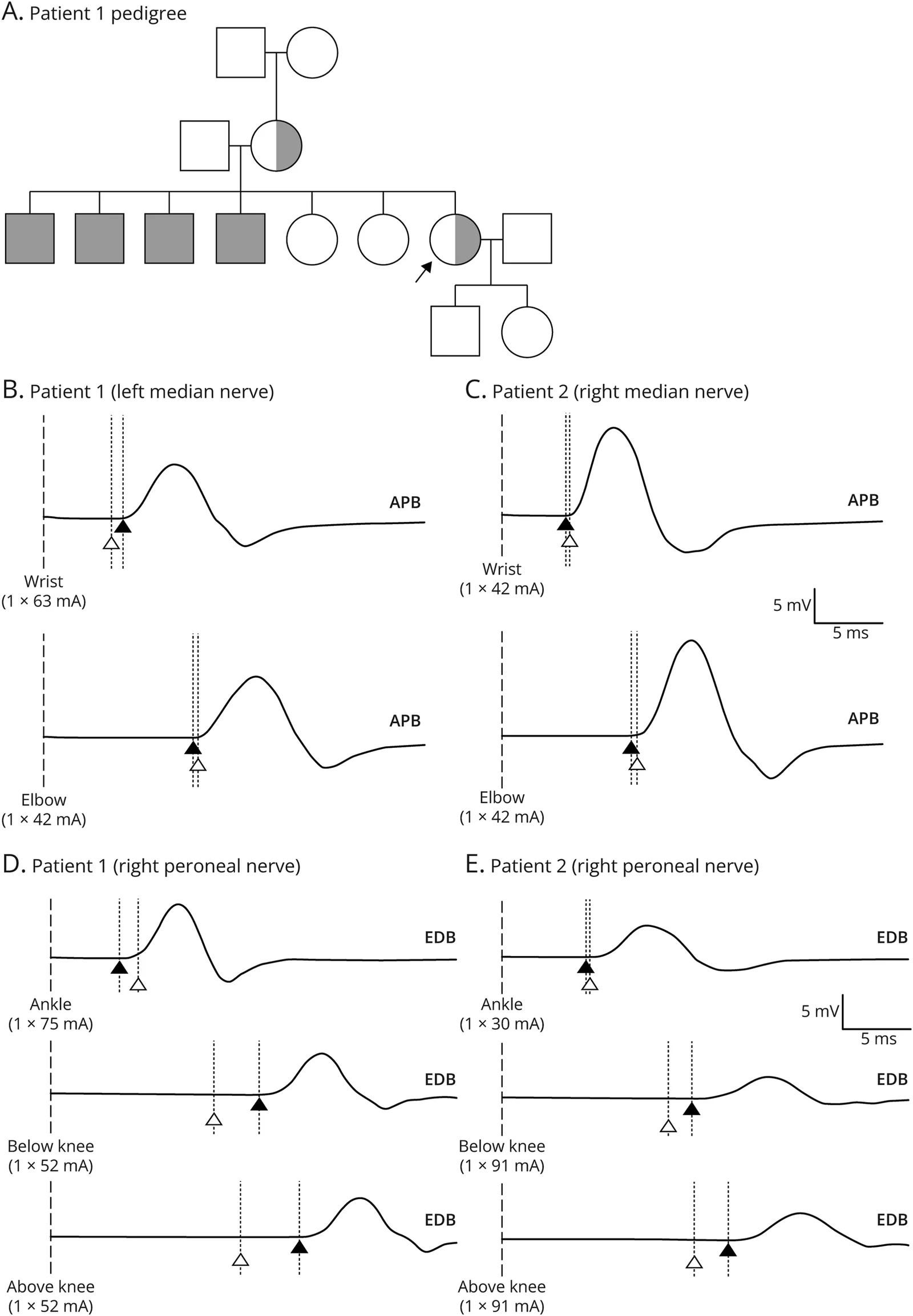
Affected Individuals and Neurophysiological Findings (A) Four-generation pedigree of the family of Patient 1 (indicated by arrow). Unfilled symbols represent unaffected family members, filled squares are affected male individuals, and half-filled circles are carrier female individuals. (B–E) Illustrative examples of the results of motor nerve conduction studies from Patient 1 (B and D) and Patient 2 (C and E). While the CMAP waveforms in D and E at more proximal points of stimulation appear dispersed with increased CMAP durations (D: distal 6.2 ms, proximal 7.7 ms, 25% increase; E: distal 7.6 ms, proximal 9 ms, 18% increase), these did not meet criteria for significant dispersion (<30%). Dashed vertical lines indicate where the stimulus artifact occurred. CMAP onsets are indicated by the dotted vertical lines associated with the filled arrowheads, whereas the dotted vertical lines associated with the unfilled arrowheads mark the upper limit of the normal range for CMAP onset, based on normal data for distal and proximal motor nerve conduction velocities.^2,3^ Calibration bars in the top row apply to the plots in A and B, whereas the calibration bars in the bottom row apply to the plots in C and D. CMAP = compound motor action potential.

On examination, there were clear optic disc margins, normal visual fields, and normal tone in all 4 limbs. However, there was mild weakness of ankle dorsiflexion on the right (Medical Research Council [MRC] grade 4). She had trouble heel walking, and there was subtle scuffing of her right shoe on the floor. Knee jerks and deep tendon reflexes in the upper limbs were physiologic, but ankle reflexes were absent. A full neurological and sensory examination was entirely normal and MRI brain and spine were normal.

In the upper limbs, nerve conduction studies (NCS) revealed slow median forearm motor nerve conduction velocity (MNCV), prolonged F-waves, and slow sensory nerve conduction velocities (SNCVs, Table and Figure B). While the results of lower limb sensory NCS and distal motor latencies (DMLs) were acceptable (Table), MNCVs were reduced (distal peroneal segment 31 m/second; proximal peroneal segment 27 m/second; tibial 30 m/second), F-responses were delayed, and compound motor action potential (CMAP) waveforms appeared dispersed (according to the reporting clinical neurophysiologist; Figure D), consistent with a demyelinating neuropathy.

**Table:** Electrophysiology Findings in Patients

Motor											
		Ulnar nerve				Median nerve				Peroneal nerve	
		DML	Velocity	Amplitude	F	DML	Velocity	Amplitude	F	DML	Velocity
Patient	Side	<3.5 ms	>50 m/second	>10 mV	<33 ms	<4.2 ms	>50 m/second	>10 mV	<32 ms	<6.0 ms	>40 m/second
1	R	3.3	50/61	19.3	34.5^a^	4.2	44^a^	17.5	35.2^a^	6.2^a^	31/39^a^
	L	NT	NT	NT	NT	NT	NT	NT	NT	8.9^a^	31/27^a^
2	R	3.9^a^	44^a^/58	13.9	32.2	4.7^a^	47^a^	11.8	34.6^a^	5.1	27/33^a^
	L	3.2	43^a^/56	12.3	32.5	5.2^a^	44^a^	11.5	33.5^a^	5.7	26/38^a^

Sensory											
			Ulnar nerve (digit V)			Median nerve (digit III)				Sural nerve	
			Velocity	Amplitude		Velocity	Amplitude			Velocity	Amplitude
Patient	Side		>50 m/second	>10 uV		>50 m/second	>10 uV			>35 m/second	>6 uV
1	R		43^a^	13		44^a^	13			42	17
	L		NT	NT		NT	NT			45	30	
2	R		48^a^	15		40^a^	11			45	5	
	L		49^a^	11		41^a^	10			50	19	

Abbreviations: DML = distal motor latency. NT = not tested.

Motor and sensory conduction studies for both patients. When 2 velocity values are displayed, the first one is distal and the second proximal (below and above the elbow for ulnar; below and above the knee for peroneal).

Amplitude values are peak-to-peak in both motor and sensory studies. The reference values are derived from the local departmental protocol, which is based on references 13 and 14.

^a^Abnormal values.

NCS in the mother were normal. Genetic testing confirmed that the patient was heterozygous for the same *PLP1* variant as her brothers (c.49_52delGCTT, p Ala17Profs*29).

In view of the genetic results, motor evoked potentials (MEPs) and somatosensory evoked potentials (SEPs) were requested to assess subclinical involvement of the corticospinal and somatosensory pathways. These provided evidence of subclinical myelopathy affecting corticospinal inputs to cervical levels on the right and the thoracic dorsal columns (details in e-Appendix).

### Patient 2

A 32-year-old woman was reported previously^4^, with only CNS involvement described (visual, somatosensory, and motor evoked potentials and neuroimaging). In summary, she presented with a slowly progressive spastic paraplegia in her 20s. Two of her brothers had been diagnosed in infancy with PMD due to a deletion of exons 5–7 of *PLP1*. Subsequently, a review of the peripheral neurophysiology data (not presented in the previous publication) revealed findings consistent with a demyelinating sensorimotor neuropathy.

The upper limb NCS showed increased median DML bilaterally and slow SNCVs. While the lower limb sensory NCS were acceptable (Table and Figure C), the lower limb MNCVs were reduced (distal peroneal segment 26 m/second and proximal peroneal segment 38 m/second), F-responses were delayed, and CMAP waveforms appeared dispersed (Figure E), with acceptable DMLs. Again, these findings are consistent with a demyelinating neuropathy.

The results of nerve conduction studies in both patients are summarized in the Table.

## Discussion

The proteolipid protein 1 gene (*PLP1*), located on the X-chromosome, encodes the PLP1 protein, the major structural protein of CNS myelin.^5^ *PLP1* is also expressed, albeit to a lesser degree, by Schwann cells in the peripheral nervous system (PNS).^6^ *PLP1* disorders are typically inherited in an X-linked manner, primarily affecting male carriers, with phenotypes ranging from spastic paraplegia 2 (SPG2) to severe forms of Pelizaeus-Merzbacher disease (PMD).^7^ While peripheral neuropathy has been identified in male individuals, it is often underappreciated in the context of more severe CNS disease.^8^

*PLP1* variants can also cause neurologic disease in female individuals with highly variable clinical phenotypes primarily affecting the CNS, including psychiatric disorders, intellectual disability,^7^ and ataxia with or without spastic paraplegia.^4^ Such clinical heterogeneity in female individuals remains incompletely understood but may be explained by variable X-chromosomal inactivation in differing cell populations within the nervous system.^9^

In this report, we describe 2 cases of *PLP1*-related neuropathy. In 1 patient, this was the cause of the presenting symptoms; in the other, neuropathy was only identified on reviewing the electrophysiology. To our knowledge, only 1 other case of *PLP1*-mediated peripheral motor-predominant neuropathy^10^ had been published. These cases highlight that *PLP1* variants in female individuals can also cause peripheral neuropathy, possibly arising from skewed X-inactivation within Schwann cells. Both patients had clinically mild or subclinical CNS involvement, and, in the absence of affected male relatives, *PLP1* variants were unlikely to have been considered (especially in Case 1). Although *PLP1* has been incorporated into the NHS Genomic Medicine Service R78 panel in the United Kingdom,^11^ it remains absent from many international testing platforms and thus may be missed despite apparent comprehensive genetic testing. Although *PLP1*-related neuropathies are likely to be rare, failure to identify them in female patients has major implications, by both preventing the opportunity for preimplantation genetic testing (significantly reducing the likelihood of transmission) and limiting access to new genetic therapies in development.^12^

*PLP1* and its alternatively spliced isoform *DM20* differ by a 35-amino-acid *PLP1*-specific domain. It has previously been demonstrated that Schwann cell expression of the full-length PLP1 isoform - but not the alternate DM20 isoform—is essential for healthy peripheral nerve function.^6^ Thus, because both patients in this study have null variants (frameshift and multiexon deletion, respectively), this will ablate both *PLP1* and *DM20* expression, consistent with this hypothesis.

We also provide further detail regarding the clinical and electrodiagnostic phenotype of the peripheral neuropathy, consistent with demyelination that is more evident in proximal nerve segments. We suggest that an appreciation of this clinical and electrophysiologic syndrome may alert clinicians to the possibility of *PLP1*-mediated motor-predominant neuropathy. Because the diagnosis can only be made by genetic testing, no additional tests such as biochemical assays are informative when making a diagnosis, and a timely genetic diagnosis may prevent potentially harmful trials of oral steroids and IV immunoglobulin.

Despite increasing availability of next-generation sequencing, many inherited neuropathy panels do not routinely include *PLP1*. We propose that *PLP1* is incorporated into appropriate genetic testing panels for undiagnosed neuropathy in female individuals presenting in their fourth and fifth decades. Identifying patients may allow access to new genetic therapies and facilitate discussion regarding preimplantation genetic testing to prevent disease transmission.
